# A study on the intervention effect of a case management model that breaks through spatiotemporal characteristics in home-based phase II exercise rehabilitation post PCI

**DOI:** 10.3389/fcvm.2024.1412675

**Published:** 2024-11-21

**Authors:** Haiqin Jin, Lingsha Wu, Ping Huang, Yeping Zheng, Yan Sun, Qin Lu, Xiaoqin Meng, Zhifang Yu

**Affiliations:** Department of Cardiology, The Second Hospital of Jiaxing, Jiaxing, China

**Keywords:** coronary heart disease, percutaneous coronary intervention, case management, cardiac rehabilitation, breaks through spatiotemporal characteristics

## Abstract

**Objective:**

This study aims to explore the effect of a case management model that breaks through the temporal and spatial characteristics on the at-home phase II exercise rehabilitation of postoperative patients treated with percutaneous coronary intervention (PCI).

**Methods:**

We used the convenience sampling method to select 103 patients with coronary artery disease (53 in the observation group and 50 in the control group) who were admitted to the Department of Cardiovascular Internal Medicine at the Jiaxing No. 2 Hospital in January 2022 and January 2023 and underwent PCI treatment as study subjects. Patients in the control group were managed by the conventional continuity of care model, and those in the observation group were managed by the case management model that breaks through the temporal and spatial characteristics. Both groups of patients were intervened and followed up for 6 months, comparing the adherence to home II exercise rehabilitation and regular follow-ups, coronary heart disease risk factor indexes, unplanned readmission rate, and the incidence of adverse cardiovascular events between the two groups of patients.

**Results:**

Exercise adherence and regular follow-up adherence of patients discharged from the hospital at 1, 3, and 6 months after PCI were higher in the observation group than in the control group (*P* < 0.05). The comparison of risk factor indicators of patients in both groups at 6 months after discharge with those of patients before discharge showed different degrees of improvement, and the difference was statistically significant (*P* < 0.05). However, the difference in fasting blood glucose in the control group at 6 months after discharge compared with that before discharge was statistically significant (*P* < 0.05), whereas there was no statistical significance in the observation group (*P* > 0.05). The incidence of major adverse cardiovascular events and unplanned readmission at 6 months after discharge between the two groups was lower in the observation group than in the control group, and the difference was statistically significant (*P* < 0.05).

**Conclusion:**

The case management mode that breaks through the spatiotemporal characteristics can improve the exercise adherence and regular follow-up adherence of post-PCI patients’ at-home phase II exercise rehabilitation, which can effectively control the indexes of body mass index (BMI), triglyceride (TG), total cholesterol (TC), and low-density lipoprotein cholesterol (LDL-C) and reduce the rate of unplanned readmission and the incidence of adverse cardiovascular events.

## 1 Introduction

Percutaneous coronary intervention (PCI) is currently the most important method of blood flow reconstruction for patients with coronary artery disease at home and abroad; however, it is not the endpoint of the treatment of coronary artery disease and does not inhibit the atherosclerotic process of the organism. Moreover, patients may still have coronary artery restenosis or even stent site after PCI (1). Studies have shown that the prognosis of post-PCI patients is closely related to their cardiac rehabilitation status (2–4), in which exercise rehabilitation is the core and main content of cardiac rehabilitation (5), and phase II rehabilitation is an out-of-hospital early rehabilitation or outpatient rehabilitation, which is the continuation of phase I rehabilitation; moreover, the foundation of phase III rehabilitation (6). Therefore, the effective implementation of phase II exercise rehabilitation is of great significance to the prognosis of patients. Although the effect of phase II outpatient rehabilitation is satisfactory, with the prolongation of discharge time and the limitation of geographical remoteness, the adherence of patients’ cardiac rehabilitation gradually decreases (7–9). Therefore, the promotion of home-based cardiac rehabilitation (CR) has a positive impact on the rehabilitation and long-term management of cardiac patients. However, the implementation of home-based exercise rehabilitation suffers from the following problems: it is difficult for patients to directly obtain one-on-one guidance from professional doctors or rehabilitation therapists, resulting in insufficient science in the development and implementation of rehabilitation programs. There is a lack of effective channels to obtain comprehensive and accurate knowledge of home exercise rehabilitation, leading to misunderstanding or blindness of patients in the process of rehabilitation. It is difficult for patients to accurately assess their exercise intensity, duration, and effect when they are rehabilitating on their own at home, and there is a lack of effective supervision mechanism. Therefore, we adopt a case management model that breaks through the characteristics of time and space to develop a professional rehabilitation program for post-PCI patients’ home-based phase II exercise rehabilitation and provide remote rehabilitation guidance, while the case manager provides effective supervision. The concept of care that breaks through the characteristics of time and space is derived from Professor Liu Rong's (10) concept of “trans-space and time medicine,” that is, the use of Internet technology to realize the implementation of medical behavior across time and across regions, breaking the restrictions of medical behavior in time and space, which is conducive to the breakthrough of patients in time and space barriers, and real-time acceptance of remote high-quality medical and nursing care. Therefore, home-based phase II exercise rehabilitation care that breaks through time and space characteristics is particularly important for post-PCI patients.

Case management, as an effective management tool for chronic diseases, is an integrated and coordinated process that involves assessment, planning, implementation, coordination, supervision, and evaluation to improve patients’ health management ability and adherence to healthcare compliance behaviors, thereby reducing healthcare costs and improving patients’ quality of life (11, 12).

Therefore, this study investigates the effect of a case management model that breaks through the temporal and spatial characteristics on adherence to home-based phase II exercise rehabilitation in post-PCI patients.

## 2 Methods

### 2.1 General information

A total of 105 patients with coronary artery disease who underwent PCI treatment at the Department of Cardiovascular Medicine of the Second Hospital of Jiaxing from January 2022 to January 2023 were selected using the convenience sampling method. Inclusion criteria included the following: (1) patients who underwent PCI for the first time successfully and had good postoperative recovery; (2) those whose risk of cardiac rehabilitation was stratified as medium–low risk (13); (3) those who had good verbal communication and reading comprehension ability; (4) those who could skillfully use a smartphone; and (5) those who voluntarily took part in this study. Exclusion criteria were as follows: (1) patients with cognitive dysfunction, communication disorders, and psychiatric disorders; (2) those with physical dysfunction who are unable to cooperate with exercise rehabilitation; (3) those with serious comorbidities that affect the judgment of the effect; (4) those who are discharged abnormally from the hospital; and (5) those who are participating in other interventional studies. Criteria for dropping out were (1) patients who could not follow up on time; (2) those who had other serious illnesses or injuries during the study period; and (3) those who requested to drop out of the study on their own initiative. Patients will be randomized in the order of their admission time: patients with early admission time will be assigned to the control group, and those with late admission time will be assigned to the observation group, in which 54 patients were included in the observation group, of which 1 patient refused to participate in the study halfway and withdrew, so a total of 53 patients were actually included. The control group included 51 patients, of which 1 patient was lost to visit, so a total of 50 patients were actually included. Comparing the general information of the two groups, the differences were not statistically significant (*P* > 0.05) and were comparable. This study was reviewed by the Medical Ethics Committee of Jiaxing Second Hospital (JXEY-2021JX072 and JXEY-2022SW083), and the patients gave informed consent.

### 2.2 Research methods

#### 2.2.1 Control group

The control group adopted conventional nursing care. These included patients who meet the criteria for the nativity and are willing to participate in the study. The nurse in charge collects the basic information of the patients through verbal questioning and reference to the medical records, makes a follow-up register, and completes the verbal health promotion, which includes the following: (1) Exercise type, including aerobic exercise (such as brisk walking, jogging, swimming, square dancing, cycling), resistance exercise (such as squatting, gluteal bridge, stretching band and dumbbells), and warm-up exercises, and finishing exercises that are mainly low-level aerobic exercise (such as full-body soft gymnastics, walking, stretching, and muscle group training). (2) Aerobic exercise frequency of three to five times a week and exercise time of 30–60 min, step by step. Resistance training according to the actual situation of patients to determine the number of times, groups, and time. (3) Exercise intensity according to the subjective fatigue scale, generally between self-perception of slightly laborious to laborious as a reasonable exercise intensity. (4) Exercise precautions (14), including measuring blood pressure and pulse within the normal range before exercise; wearing appropriate clothing and shoes during exercise; not doing strenuous exercise 1–2 h after meals; adjusting the amount of exercise according to the season; stopping exercise immediately if there is discomfort such as chest tightness, chest pain, and palpitations; and seeking medical attention in time if it persists without relief. The above content was made into a health education leaflet and distributed to the patients and, at the same time, made into a QR code that was scanned to WeChat and saved for patients to check at any time. The patients were told that they would receive follow-up calls at 2 weeks, 1 month, 3 months, and 6 months after discharge and that they would receive follow-up calls at 1, 3, and 6 months after discharge, and the follow-up visits would include participation in exercise, including the type, frequency, and time of exercise, and whether there were any abnormalities.

#### 2.2.2 Observation group

The patients were intervened with the case management model that breaks through spatiotemporal characteristics. By summarizing the health behavior problems of the patients discharged from the hospital after PCI and consulting with the chief physician of the cardiology department and nursing specialists, we clarified the duties and work content of the case manager and constructed the case management model that breaks through spatiotemporal characteristics, as shown in Table 1.

**Table 1:** A case management model intervention program that breaks through temporal and spatial characteristics.

Intervention step	Intervention content
Conclude cases	1. The case manager communicates and exchanges with the patients and their families in depth to establish a sense of trust, informs them of the purpose of the study and what they need to cooperate with to participate in the study, and then incorporates them into the case management and establishes a personal file after informed consent.
	2. We distributed the Cardiac Rehabilitation Handbook to the patients and their families, informing them of the importance of home-based phase II cardiac rehabilitation and the precautions to be taken.
Assessment	3. The case manager interviews and evaluates the patient's condition, dietary habits, psychosocial, work and rest, exercise habits, and willingness to exercise through the motivational interviewing method to identify the patient’s existing and potential problems and clarify the direction of improvement.
	4. The case manager assesses the patient's exercise fitness and develops an individualized exercise prescription based on a 6-min walk test performed by the cardiac rehabilitation therapist.
Planning	5. The case manager trains and guides the intake patients and their families on disease-related knowledge and self-management skills.
	6. The case manager identifies the patient's problem, organizes and coordinates case management team meetings, and participates with the patient and family in the development of an individualized intervention plan, including medication, diet, exercise, psychological adjustment, smoking cessation, and follow-up visits.
Conduct	7. The case manager and the patient or family met face-to-face or via WeChat and leave each other telephone contact information.
	8. The case manager monitors the patient's adherence to at-home phase II exercise rehabilitation based on the patient’s personalized exercise prescription, breaking through time and space constraints through mobile Internet technology. Patients are recommended to wear exercise bracelets when exercising to monitor the target heart rate and ensure safety. Recommend patients to use exercise app or WeChat applets, such as KEEP, Huawei Sports, and WeChat Sports to record exercise time and exercise volume. Patients are advised to clock in the WeChat app to record after exercise. The case manager will check the patient's exercise status at least once a day, and timely WeChat or telephone intervention will be carried out when problems are found. When the patient encounters problems during the rehabilitation process, he/she can contact the case manager at any time via WeChat or telephone, and if the case manager is unable to solve the problem, he/she will contact and communicate with the relevant staff to solve the problem together.
	9. Case managers assist patients in paying attention to the “Nanhu Cardiology” WeChat public number, and the case management team pushes out coronary heart disease-related health knowledge from time to time, mainly in the form of graphics and videos, which are reviewed and published by the Director of the Department of Cardiovascular Medicine. The case manager will also push the health-related knowledge through WeChat according to the patient’s specific situation.
	10. The case management team developed emergency procedures and contingency plans for various emergencies through a literature search and consultation with the chief cardiovascular physician and nursing experts to ensure the safety of phase II rehabilitation for post-PCI patients.
	11. At 2 weeks, 1 month, 3 months, and 6 months after discharge, the case manager conducted follow-up visits via WeChat, which included patients’ compliance in taking medication on time, eating healthy, exercising as required, psychological adjustment, smoking cessation, and follow-up review on time.
Estimation	12. Patients are asked to send their cardiac rehabilitation status to the case manager's WeChat, which is then recorded in the patient's file by the case manager.
Send back	13. Provide timely feedback on the patient's documented cardiac rehabilitation and make timely adjustments to the original program based on follow-up visits.
Wind up cases	14. Patients were discharged from the hospital at 1, 3, and 6 months to collect the evaluation indicators and end the intervention.

#### 2.2.3 Flowchart

**Figure 1:**
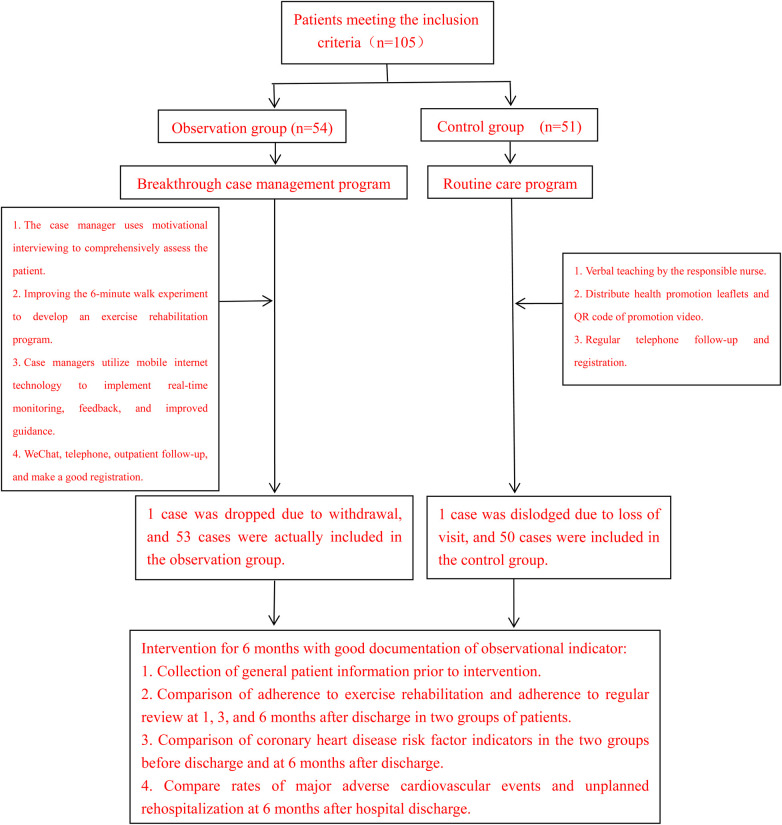
Flowchart.

The inclusion and exclusion criteria for the sample and the entire study protocol are shown in Figure 1.

### 2.3 Observation indicators

(1) Post-PCI patients’ phase II rehabilitation exercise adherence and regular review adherence: Adherence was evaluated at 1, 3, and 6 months after discharge as evaluation nodes. Exercise adherence means that patients perform rehabilitation exercises according to the corresponding requirements. Among them, the patients in the control group exercised as required according to the health education leaflet, and the researcher followed up with their family members by phone to know the patients’ exercise adherence. The patients in the observation group exercised according to their own personalized exercise prescription, and the researcher monitored the patients’ exercise adherence through WeChat punch cards. aerobic exercise less than 3 times per week for <30 min each time was judged as poor adherence to exercise (15). Adherence to review means that the patient comes to the hospital for regular reviews as required. The investigators monitored the adherence of both control and observation group patients by reviewing their visit records or by the patients themselves providing their out-of-hospital review records. Poor compliance with the review was judged if there was a failure to comply with the required review (16).
(2) Coronary heart disease risk factor indicators: body mass index (BMI), fasting blood glucose (FBG), triglyceride (TG), total cholesterol (TC), and low-density lipoprotein cholesterol (LDL-C) were tested before intervention and at 6 months.
(3) Incidence rate of major adverse cardiovascular events and unplanned rehospitalization rate: By reviewing the patients’ consultation and follow-up records, we found out whether there were any adverse cardiovascular events within 6 months of discharge, mainly including the incidence of non-fatal myocardial infarction, revascularization, angina pectoris, heart failure, and sudden cardiac death.

### 2.4 Quality control

(1) Prior to the intervention, participants were screened according to strict inclusion and exclusion criteria and were randomized into groups to reduce bias.
(2) Prior to the study, we were uniformly trained and assessed on the correct completion of the questionnaire, and information and data were collected using blinded methods. Blinding is reflected in the fact that the informants and data collectors were not aware of the subgroups of patients to minimize information bias in the information collection and data collection process.
(3) Information was double checked to ensure the accuracy of data entered.
(4) The patient's 6-min walk test was an exercise fitness assessment performed by a cardiac rehabilitation therapist in strict accordance with the requirements associated with the trial, and the data were all true and valid.

### 2.5 Statistical methods

The data were analyzed by SPSS 25.0 statistical software. The statistical description of the measurement data was expressed as (±*s*). The two-sample paired mean *t*-test was used for the measurement data in the same group, and the independent samples *t*-test was used for the measurement data between the two groups. The counting data were expressed as cases and percentages. *χ*^2^ test was used, and the Fisher exact probability test was used when at least one theoretical frequency was <1. The test level *α* = 0.05.

## 3 Results

There was no statistically significant difference between the general clinical data of the two groups of patients (*P* > 0.05), as shown in Table 2.

**Table 2:** Comparison of general clinical data between the two groups.

Event	Observation group (*n* = 53)	Control group (*n* = 50)	*x*^2^/*t*	*P*
Age (years, $\bar{x}\pm s$)	56.66 ± 11.509	60.43 ± 9.519	1.817	0.072
Gender (*n*)				
Male	44	34	3.157	0.076
Female	9	16		
Education (*n*)				
Primary school students and below	13	16	2.555	0.466
Middle school students	27	18		
Senior high school students	7	10		
College students and above	8	6		
Complication (*n*)				
None	2	3	2.224	0.527
1	10	12		
2	6	9		
≥3	35	26		
Number of vascular lesions (*n*)				
1	20	14	1.245	0.537
2	18	18		
≥3	15	18		
Number of sent implants (*n*)				
1	45	40	1.141	0.565
2	6	9		
≥3	2	1		

Comparing the exercise compliance and review compliance of the two groups of patients, those of the observation group were higher than the control group, and the difference was statistically significant (*P* < 0.05), as shown in Table 3.

**Table 3:** Comparison of exercise adherence and review adherence between the two groups.

Group	Exercise compliance			Review compliance		
	1 month after discharge	3 months after discharge	6 months after discharge	1 month after discharge	3 months after discharge	6 months after discharge
Observation group (*n* = 53)	50 (94.3)	51 (96.2)	51 (96.2)	53 (100.0)	50 (94.3)	50 (94.3)
Control group (*n* = 50)	40 (80.0)	38 (76.0）	36 (72.0)	42 (84.0)	40 (80.0)	40 (80.0)
*χ* ^2^	4.797	8.962	11.509	9.194	4.797	4.797
*P*	0.029	0.003	0.001	0.020	0.029	0.029

It can be seen that both intervention types led to a decrease in the indicators of coronary heart disease risk factors such as BMI, TG, TC, LDL-C, and fasting blood glucose for patients at 6 months after discharge compared to before discharge. The difference in TG, TC, and LDL-C between the two groups of patients at 6 months after discharge compared with before discharge was statistically significant (*P* < 0.05). The difference in the BMI of the observation group between 6 months after discharge and before discharge was statistically significant (*P* < 0.05), while that of the control group was not statistically significant (*P* > 0.05). The fasting blood glucose in the control group 6 months after discharge compared with the pre-discharge difference was statistically significant (*P* < 0.05), while that of the observation group was not statistically significant (*P* > 0.05). Specific details are shown in Table 4.

**Table 4:** Comparison of coronary heart disease risk factor indicators between the two groups $\left(\bar{x}\pm s\right)$.

Event		Observation group (*n* = 53)	Control group (*n* = 50)	*t*	*P*
BMI (kg/m^2^)	Pre-hospitalization	25.35 ± 2.909	24.23 ± 2.896	1.938	0.055
	6 months after discharge	23.95 ± 2.509^a^	24.06 ± 3.245	0.190	0.850
TG (mmol/L)	Pre-hospitalization	1.90 ± 1.675	1.72 ± 0.876	0.680	0.498
	6 months after discharge	1.37 ± 0.620^a^	1.74 ± 0.826^a^	2.371	0.020
TC (mmol/L)	Pre-hospitalization	4.40 ± 1.236	4.13 ± 1.185	1.127	0.262
	6 months after discharge	3.41 ± 0.614^a^	3.76 ± 0.920^a^	2.104	0.038
LDL-C (mmol/L)	Pre-hospitalization	2.53 ± 1.055	2.31 ± 1.057	1.003	0.318
	6 months after discharge	1.61 ± 0.515^a^	1.99 ± 0.899^a^	2.386	0.020
Fasting blood glucose (FBG) (mmol/L)	Pre-hospitalization	6.20 ± 2.130	6.63 ± 2.552	0.909	0.366
	6 months after discharge	6.00 ± 1.619	6.04 ± 1.250^a^	0.133	0.895

^a^ *P* < 0.05 for 6 months of discharge compared to pre-discharge.

When comparing the incidence of major adverse cardiovascular events and unplanned readmission at 6 months after discharge between the two groups, the observation group was lower than the control group, and the difference was statistically significant (*P* < 0.05), as shown in Table 5.

**Table 5:** Comparison of the incidence of major adverse cardiovascular events between the two groups of patients.

Group	Unplanned readmissions (*n*)	Adverse cardiovascular events					
		Non-fatal myocardial infarction (*n*)	Non-lethal hemodialysis (*n*)	Angina pectoris (*n*)	Heart failure (*n*)	Cardiac death (*n*)	Total incidence (%)
Observation group (*n* = 53)	0	0	0	0	0	0	0
Control group (*n* = 50)	4 (8.0)	1 (2.0)	1 (2.0)	3 (6.0)	2 (4.0)	0	7 (14.0)
*P*	0.006	0.001					

## 4 Discussion

A case management model that breaks through time and space can improve the exercise compliance and review compliance of post-PCI patients. The results of this study showed that the exercise compliance and review compliance of patients in the observation group were higher than those in the control group at 1, 3, and 6 months after the 6-month intervention, which is consistent with the results of many studies at home and abroad (17–19). In addition, Antoniou et al. (20) found that home-based exercise rehabilitation did not increase the incidence of exercise-related adverse events. Keteyian et al. (21) demonstrated that the telehealth CR model is similar in training intensities to the conventional outpatient CR in coronary artery disease (CAD) patients with low to moderate cardiovascular risk. It can be seen that this type of management mode through mobile Internet technology and a remote monitoring system combined with multidisciplinary, personalized, and programed management mode has improved the participation and review rates of cardiac rehabilitation for patients. Exercise-based cardiac rehabilitation is recognized worldwide as a key component for the comprehensive management of coronary heart disease. Moreover, Dibben et al. (5) found that exercise rehabilitation effectively reduces mortality, hospitalization, and myocardial infarction rates due to cardiovascular disease, and that relative to the non-participating coronary heart disease population, the risk of cardiovascular death in the participating exercise rehabilitation group decreased by 26%, the risk of cardiac readmission decreased by 23%, and the risk of having a myocardial infarction decreased by 18%. In this study, the exercise compliance of patients in the control group decreased significantly with the prolongation of the discharge time (22, 23), while the exercise compliance of patients in the observation group did not see a significant decrease with the prolongation of the discharge time, which may be attributed to the fact that the case manager conducted a comprehensive assessment of the patients and fully explained the knowledge related to the disease at the time of case intake, to make the patients clearly understand the hazards of the disease and the importance of exercise rehabilitation and regular follow-up on the improvement of the prognosis of the disease, making the patients recognize and accept exercise rehabilitation from the ideology. At the same time, through in-depth communication and exchange, trust and affection are built. When formulating the plan, the case manager used motivational interviewing to assess the patient's willingness to participate in exercise rehabilitation and to identify the actual difficulties they faced in doing so (24). Based on this information and combined with the results of the 6-min walk test, the case manager collaborated with the patient, the doctor, and the rehabilitator to formulate a personalized exercise rehabilitation plan to encourage the patient to take participate in exercise rehabilitation. In this study, 11 patients in the observation group changed their exercise prescription, 2 of them after 1 month of exercise and the remaining 9 after 3 months of exercise. After the patients were discharged from the hospital, the case manager supervised their adherence to exercise rehabilitation through the Internet technology and the remote monitoring system to break through the spatial and temporal characteristics.

A time-breaking case management model improves the indicators of relevant coronary heart disease risk factors in post-PCI patients. Obesity, hyperlipidemia, and hyperglycemia are not only important risk factors for coronary heart disease but also have a significant impact on patient prognosis. It has been shown that obese patients with coronary heart disease have a significantly increased risk of reinfarction and total mortality, which are independent risk factors for coronary heart disease (25). In obese patients, skeletal muscle blood flow is reduced, leading to decreased glucose uptake by skeletal muscle cells. This reduction enhances insulin resistance in target organs, leading to endothelial cell dysfunction and accelerating the process of atherosclerosis (26, 27). Dyslipidemia is a major risk factor for coronary heart disease, and hypercholesterolemia is involved in increasing oxidative stress, mitochondrial dysfunction, and inflammation triggering apoptosis, leading to myocardial dysfunction and myocardial infarction. Elevated serum LDL-C levels are a major risk factor for the development of ischemic heart disease (28). In diabetic patients, platelet and endothelial dysfunction leads to lipid deposition in atheromatous plaques, causing negative arterial remodeling and eventual occlusion of blood vessels (29). The results of this study showed that BMI, TG, TC, LDL-C, and fasting blood glucose decreased in both groups of patients following the intervention. Notably, the BMI of the observation group decreased significantly compared to that of the control group, with a statistically significant difference observed before and after the intervention (*P* < 0.05). In contrast, the BMI index of the control group decreased slightly, with no statistically significant difference before and after the intervention (*P* > 0.05). Furthermore, TG, TC, and LDL-C decreased significantly in the observation group compared to the control group through the intervention, and the difference was statistically significant (*P* < 0.05). These findings are consistent with the results of Ögmundsdóttir Michelsen et al. (19). This may be due to the time-breaking case management model led by the case manager, which was combined with multi-departmental and multidisciplinary personalized full and comprehensive management of the patient from case intake to closure. This approach, in addition to exercise rehabilitation interventions, also involves comprehensive assessment and interventions, such as medication, diet, and psychology, and the process of intervention lasts for the patient's entire study period. In contrast, the patients in the control group relied only on the researcher to conduct routine health investigation after a comprehensive assessment before discharge, which lacked pertinence and personalization, thus potentially affecting patient acceptance. At the same time, the patients in the control group received telephone follow-ups of 1, 3, and 6 months after discharge and lacked real-time intervention and supervision by professionals after discharge, so their healthy behavioral habits could not be sustained, and they resumed their bad living habits with the extension of time. However, the decrease in fasting blood glucose of patients in the observation group was not as obvious as those in the control group, which may be due to several factors affecting blood glucose fluctuations, such as the dawn phenomenon, where the secretion of growth hormone and other glucose-raising hormones such as cortisol during the night leads to normal blood glucose levels at midnight but elevated fasting glucose in the morning. In addition, the Somogyi phenomenon can occur when patients suffer from frequent hypoglycemia at night, resulting in rebound hyperglycemia the next morning, which reflects the body’s self-regulation of blood sugar. In such cases, when blood sugar levels drop, the body responds by increasing glucagon, converting glycogen to glucose, and thereby raising blood sugar levels.

A time-breaking case management model reduces the rate of unplanned rehospitalization and the incidence of major adverse cardiovascular events in patients. The results of this study showed that the rates of unplanned in-hospitalization and major adverse cardiovascular events were lower in the observation group compared with the control group 6 months after the intervention, and the difference was statistically significant (*P* < 0.05) with no cardiac deaths in both groups. Cardiac rehabilitation is the focus of non-pharmacological treatment and secondary prevention of coronary heart disease, and exercise rehabilitation is its core content. Cardiac rehabilitation can improve patients’ exercise tolerance, improve clinical symptoms, and reduce disability and mortality. However, there are certain risks in cardiac rehabilitation, which are mainly related to the fact that the process of exercise rehabilitation increases myocardial oxygen consumption and decreases coronary perfusion. In this study, the time-breaking case management model used 6-min walk observation to assess patients’ exercise fitness and also assessed patients’ exercise habits, so that the patients, case managers, and doctors worked together to formulate personalized exercise prescriptions. The patients are supervised through Internet technology and a remote monitoring system after they were discharged from the hospital to ensure their safety during exercise. This is consistent with the results of several studies at home and abroad (17–19). Moreover, the case manager dynamically monitors the patient's exercise ability and follows up to change the exercise prescription in time to ensure the effectiveness of exercise rehabilitation. Therefore, unplanned readmission rates and the incidence of adverse cardiovascular events in patients were greatly reduced. This is consistent with the findings of Yang et al. (30) and Joo and Huber (31).

Despite the rigorous design of this study, there are some limitations: (1) The collection of information on some variables was done using a homemade scale, which may have been subject to a slight measurement bias, potentially affecting the results. (2) This study was conducted within the same department of a hospital; however, communication between patients was not excluded. (3) Participants were free to seek additional medical care from other healthcare providers, and it is not certain whether this would have had an impact on the results of the study. (4) Due to time and human resource constraints, data were only collected up to 6 months after patients were discharged from the hospital. (5) The data came from the same hospital and lacked generalizability.

## 5 Conclusion

The time- and space-breaking case management model can improve exercise adherence and review adherence in post-PCI patients, improve the relevant coronary heart disease risk factor indicators in post-PCI patients, and reduce the rate of unplanned rehospitalization and major adverse cardiovascular events in patients. This management model can also be applied to other chronic diseases to improve patients’ quality of life and compliance behavior and reduce healthcare costs. Therefore, it is suggested that this model of care can be promoted and disseminated in other hospitals and can also be piloted for other chronic diseases.

## Data Availability

The original contributions presented in the study are included in the article/Supplementary Material, further inquiries can be directed to the corresponding author.
